# Plasma Processing of Low Vapor Pressure Liquids to Generate Functional Surfaces

**DOI:** 10.3390/molecules25246024

**Published:** 2020-12-19

**Authors:** Sandra Gaiser, Urs Schütz, Patrick Rupper, Dirk Hegemann

**Affiliations:** Empa, Swiss Federal Laboratories for Materials Science and Technology, Plasma & Coating Group, Lerchenfeldstrasse 5, 9014 St. Gallen, Switzerland; urs.schuetz@empa.ch (U.S.); patrick.rupper@empa.ch (P.R.)

**Keywords:** plasma polymerization, liquid substrate, coating of liquids, solid-liquid gradient, surface functionalization, polyethylene glycol

## Abstract

The concept of depositing solid films on low-vapor pressure liquids is introduced and developed into a top-down approach to functionalize surfaces by attaching liquid polyethylene glycol (PEG). Solid-liquid gradients were formed by low-pressure plasma treatment yielding cross-linking and/or deposition of a plasma polymer film subsequently bound to a flexible polydimethylsiloxane (PDMS) backing. The analysis via optical transmission spectroscopy (OTS), optical, confocal laser scanning (CLSM) and scanning electron microscopy (SEM), Fourier transform infrared (FTIR) and X-ray photoelectron spectroscopy (XPS) as well as by water contact angle (WCA) measurements revealed correlations between optical appearance, chemical composition and surface properties of the resulting water absorbing, covalently bound PEG-functionalized surfaces. Requirements for plasma polymer film deposition on low-vapor pressure liquids and effective surface functionalization are defined. Namely, the thickness of the liquid PEG substrate was a crucial parameter for successful film growth and covalent attachment of PEG. The presented method is a practicable approach for the production of functional surfaces featuring long-lasting strong hydrophilic properties, making them predestined for non-fouling or low-friction applications.

## 1. Introduction

The deposition of thin films on solid substrates via low-pressure processes is a well-known approach for a wide range of applications. A less explored and challenging field is the formation of such films on the surfaces of low-vapor pressure liquids. In this paper we want to give a detailed summary of the research which has already been conducted in this area. Based on these ideas, we subsequently present our novel approach for the plasma processing of liquids allowing not only the formation but simultaneous functionalization of thin films on liquid substrates. The first attempt to coat liquids was made by Ye et al., who successfully deposited continuous metallic films on silicone oil by RF magnetron sputtering and investigated the nucleation and growth mechanisms taking place during film formation [1,2,3]. Other groups followed this approach using different kinds of liquid substrate materials, such as silicone oils, ionic liquids and vegetable oils, to synthesize metal films and nanoparticles made of gold, silver, titanium, aluminum, iron, nickel and copper via sputtering and thermal evaporation processes [4,5,6,7,8,9,10,11,12,13,14,15,16,17,18,19,20,21,22,23,24,25,26,27]. Besides, the growth of crystals from different aromatic hydrocarbons, zinc, copper and gold on silicone oil and ionic liquids by vapor phase deposition was studied [28,29,30,31,32,33,34,35]. It is unanimously reported that a multi-step formation mechanism occurs when depositing metals on liquids [1,2,3,4,5,7,8,9,10,13,15,16,17,18,19,21,23,24,25,26]. First, nucleation and growth of smaller clusters can be observed. Those clusters freely float on the liquid’s surface and gradually form bigger aggregates. Finally, closed, free standing films are obtained which feature characteristic, regular microstructures or cracks which exhibit surprising similarities for the examined materials [1,2,5,6,7,8,9,10,11,15,16,17,18,19,20,27]. Internal film stress is discussed as the main reason for the appearance of microstructures, wrinkles and cracks [6,7,8,9,10,11,15,16,17,18,19,20,27].

Keppner et al. were the first to present a method to cover a liquid with a thin polymer film by means of a low-pressure deposition process [36]. Based on this work it was shown that via (initiated) chemical vapor deposition, ((i)CVD) polymer coatings consisting of parylene can successfully be deposited on liquids such as glycerin, liquid paraffin and silicone oil, resulting in closed membranes encapsulating the liquid substrate material. In contrast to metal films, the deposition of parylene resulted in transparent and stable yet extremely flexible films. Other polymer materials, such as poly(2-hydroxyethyl methacrylate) (PHEMA), were deposited via iCVD as films, encapsulations or nanoparticles on ionic liquids, silicone oils or liquid PDMS [37,38,39,40,41,42]. The mechanisms which are discussed to be responsible for film and particle formation at the liquid’s surface are similar to the processes described for the deposition of metals. The authors describe an initial nucleation and subsequent formation of bigger aggregates due to film-forming material randomly diffusing across the liquid’s surface [40,41,42]. Whether the precursor material arriving at the liquid substrate forms floating clusters or submerges into the liquid depends on the intermolecular forces predominating gravitational effects [27,41,42,43]. In accordance with the deposition of metal films, the occurrence of internal film stress and resulting wrinkling has been described for plasma-enhanced chemical vapor deposition (PECVD) processes which have been applied using monomers, such as ethylene, methane, hexamethyldisiloxane (HMDSO), silane or a mixture of carbon tetrafluoride and argon, to deposit closed films on liquids [27,43]. If non-film-forming process gases such as argon and hydrogen are used for the plasma treatment of liquids, such as PDMS, the liquid’s surface gets cross-linked and solidified. Thus, thin, free-standing membranes made of silicone can be produced [27,43].

Those methods to grow films on liquids exhibit promising potential for various applications. Examples include the encapsulation of liquids for the production of optical microdevices, such as liquid waveguides and tunable microlenses, and for drug delivery systems, and the coverage of micro-channels to obtain micro-structures made of the film-forming material [27,37,38,39,44,45,46,47]. Such thin films may also serve as biocompatible membranes or can be used for the fabrication of sensors or mirrors [14,48,49].

**Figure 1:**
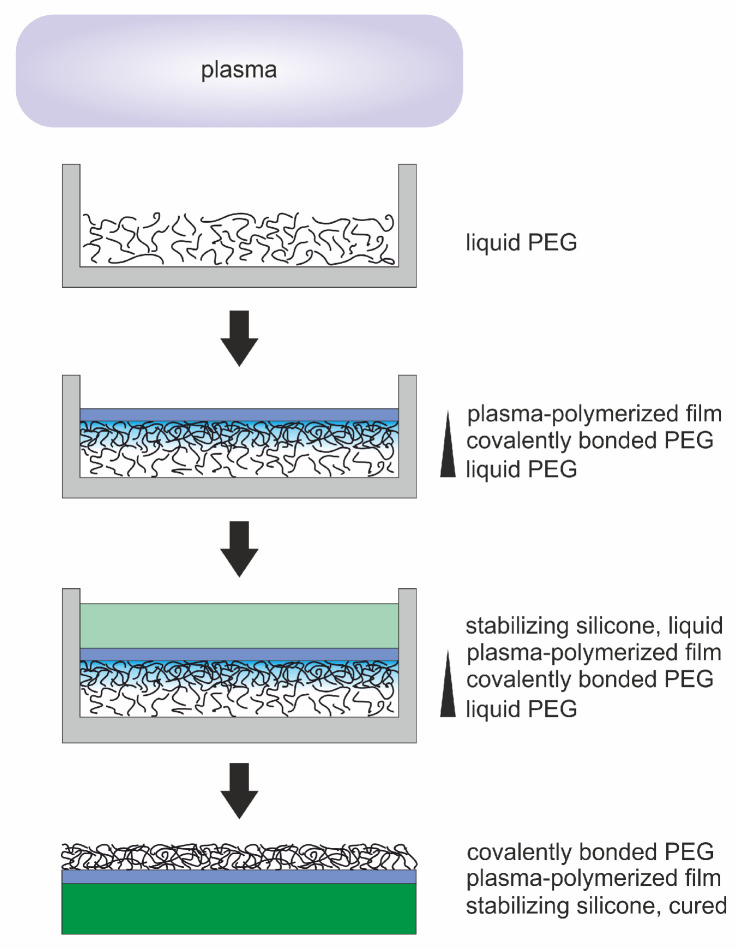
Step-by-step depiction of sample preparation. Plasma treatment of liquid polyethylene glycol (PEG) held in a Petri dish leads to cross-linking of molecular chains and the deposition of an a-C:H film. To stabilize the plasma-polymerized film, it is covered with liquid polydimethylsiloxane (PDMS). After curing, the sample can be removed from the Petri dish. Washing away excess PEG leaves only covalently bonded PEG on the sample’s surface.

So far, the described approaches have aimed at depositing solid materials on liquids while using the liquid just as supporting substrate. Going a step further, we proposed a novel approach to use a plasma process to deposit free-standing, thin films on liquid polyethylene glycol (PEG) and to additionally functionalize these films by covalently attaching PEG molecules [50]. The process schematic is depicted in Figure 1. Low-pressure plasma processes produce UV radiation, radicals and energy-rich ions which are able to cause a cross-linking of liquid molecules between the liquid and the solid coating. During film formation, the liquid is shielded more and more by the growing film, and the degree of cross-linking can be expected to decrease towards the bottom of the liquid volume. This leads to a covalent bonding of PEG molecules to the solid film but still leaving mobile, "liquid" molecular chains on the lower side of the sample. In this way, solid-liquid vertical gradients can be produced by equipping thin films with specific surface features originating from the liquid material. By covering the solid film with liquid silicone (polydimethylsiloxane (PDMS)), which was cured afterwards, we were able to stabilize the samples and lift them off. In this way, the formerly inaccessible PEG side could be turned upwards and was analyzed. We could verify an attachment of PEG resulting in highly hydrophilic surface properties [50].

Besides its hydrophilic character, PEG is non-toxic and biocompatible and is therefore used in many biomedical, pharmaceutical and industrial applications [51,52,53,54]. Its ability to form hydrogen bonds and hydrated layers in aqueous environments, the mobility of its molecular chains and the resulting steric hindrance effects lead to great potential for non-fouling surfaces resisting the adsorption of cells and proteins [52,53,54,55,56,57,58,59,60,61,62].

Methods which have been reported for the immobilization of PEG usually require multi-step processes which are time-consuming, costly and harmful to the environment [54,63,64,65,66]. Furthermore, those PEG coatings lack stability, especially in aqueous environments, which is a big disadvantage for many applications [55,67,68]. Low-pressure plasma processes are favorable with their ability to covalently bond molecular chains of liquid PEG to a surface [64,69]. However, the plasma may cause fragmentation of the PEG molecules, which can thus loose their hydrophilic properties [68,70]. To maintain a good non-fouling ability of surfaces, it is crucial to maintain the polyether structure of PEG [70]. With our top-down approach using PEG as a substrate material which is subsequently covered with a plasma-polymerized film (PPF), this loss of functionality can be prevented.

We used two different plasma processes for the production of the samples presented in this work. By plasma processing using argon and oxygen, partial cross-linking due to impinging ions, radicals and UV radiation occurred at the liquid’s surface, forming a solid PEG film. By applying an ethylene plasma, we combined the cross-linking with the effect of plasma polymerization, leading to a thin (∼200 nm [50]) hydrocarbon film covering the liquid and being covalently bond to it. Results on the composition, structure and surface properties of these samples and mechanisms leading to stable functionalized surfaces are presented and discussed. The samples were analyzed by optical transmission spectroscopy (OTS), optical, confocal laser scanning (CLSM) and scanning electron microscopy (SEM), Fourier transform infrared spectroscopy (FTIR), X-ray photoelectron spectroscopy (XPS) and water contact angle (WCA) measurements.

The samples prepared by using an argon–oxygen plasma and an ethylene plasma will be called Ar-O${}_{2}$ samples and Ar-C${}_{2}$H${}_{4}$-Ar samples, respectively. The plasma-polymerized amorphous hydrocarbon (a-C:H) film which is formed by applying an ethylene plasma will be abbreviated with PPF. The words silicone and PDMS will be used interchangeably.

## 2. Results

### 2.1. Optical Transmission Spectroscopy

**Figure 2:**
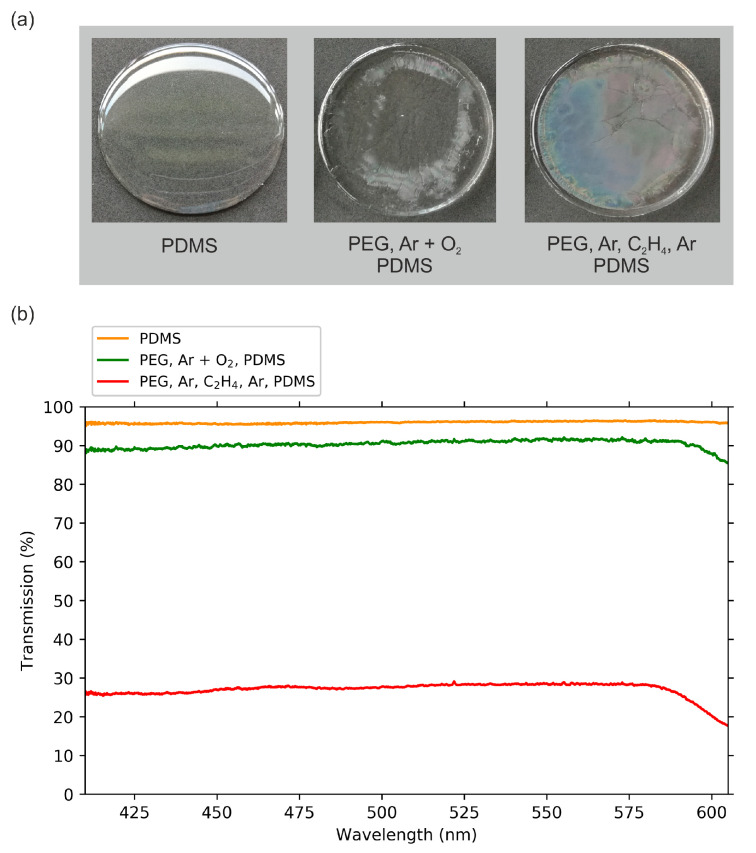
(**a**) Photographs and (**b**) optical transmission spectra of pure PDMS and plasma-treated PEG with PDMS backing. The samples had been lifted off the Petri dishes.

The first method to analyze the samples was optical inspection. A clear difference could be seen concerning the transparency, as shown in Figure 2a. Compared to pure, cured PDMS, which seems to be completely transparent, the Ar-O${}_{2}$ samples show matte regions with lines appearing to be cracks or wrinkles. The Ar-C${}_{2}$H${}_{4}$-Ar samples have a closed matte surface. However, they also feature cracks and inhomogeneities. These properties turned out to be well-reproducible when using the described plasma processes. To quantify the changes in surface appearance in comparison to pure PDMS, we investigated the differences in optical transmission of the samples. For this purpose, optical transmission spectra were recorded. For each spectrum shown in Figure 2b an average of eight spectra were recorded, each measured on a different spot, considering the proportional presence of more transparent and more matte surface areas. Thus, the presented results represent mean transmission values for each sample.

The three samples, pure silicone (PDMS) and the Ar-O${}_{2}$ and Ar-C${}_{2}$H${}_{4}$-Ar samples, reduced the intensity of the transmitted light due to scattering effects. In accordance with the optical impression (Figure 2a), the PDMS sample was rather transparent and still transmitted 96% of the light. For the Ar-O${}_{2}$ samples with their non-uniform surface, the transmission depended on the position whereat the measurement was conducted. The presented mean transmission value shows a reduction in light intensity by 9% compared to the white light spectrum. The silicone backing contributed about 50% to this attenuation. There were still quite transparent regions visible on the sample which were comparable with PDMS. The strongest reduction, however, could be measured for a matte region on the sample’s surface where 86% of the original light was transmitted. A further, much stronger attenuation could be found for the Ar-C${}_{2}$H${}_{4}$-Ar sample: On average, only 28% of the light was transmitted. The values scattered between 17% and 30.5%. This illustrates that this plasma-processed PEG surface still lacked homogeneity. The drop in the spectra towards higher wavelengths shows that these surfaces transmit light in the visible range selectively, which corresponds with the optical appearance of some samples featuring a bluish color.

During the development of the sample preparation process, it turned out that successful plasma processing of the liquid leading to a cross-linked, matte surface depends strongly on a thin and uniform distribution of the liquid PEG film in the Petri dish. It could be observed that rather thick PEG films inhibited film growth on the liquid’s surface. To investigate this effect, we subsequently prepared samples with different PEG filling levels. A well-defined amount of liquid PEG was poured in Petri dishes followed by upturning them for a selected time to drain parts of the liquid.

**Figure 3:**
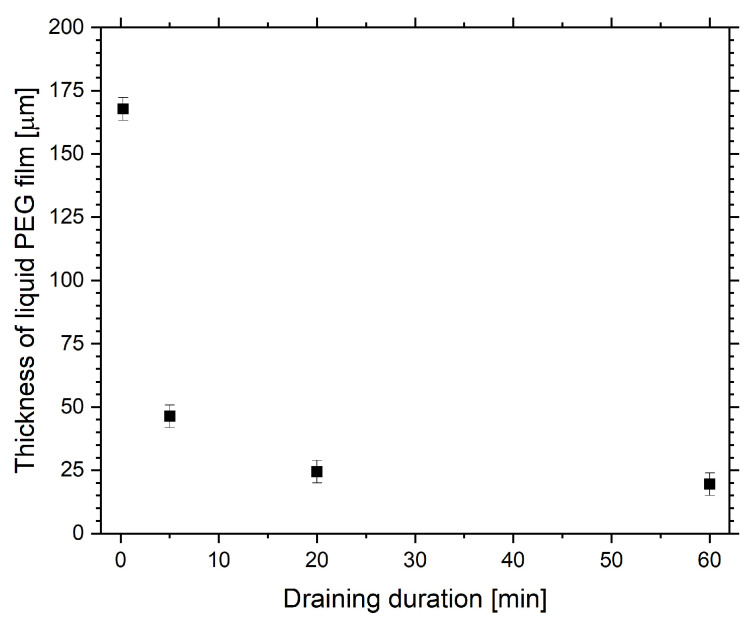
Dependence of the thickness of the liquid PEG substrate on the draining duration during sample preparation.

To get an idea about the actual filling level of the liquid substrates, the approximate heights of the PEG layers were derived. Initially, 2.5g of PEG was poured into the Petri dishes. After draining for different time periods, the remaining mass was determined by weighing. With the density of PEG (1.123 g/cm${}^{3}$ [71]) and the diameter of the Petri dish, the height of the PEG substrate depending on the draining duration could be calculated. The results, displayed in Figure 3, show a maximum height of 167.7± 4.5 μm. When drained for one hour, the thickness was reduced to 19.5± 4.5 μm.

**Figure 4:**
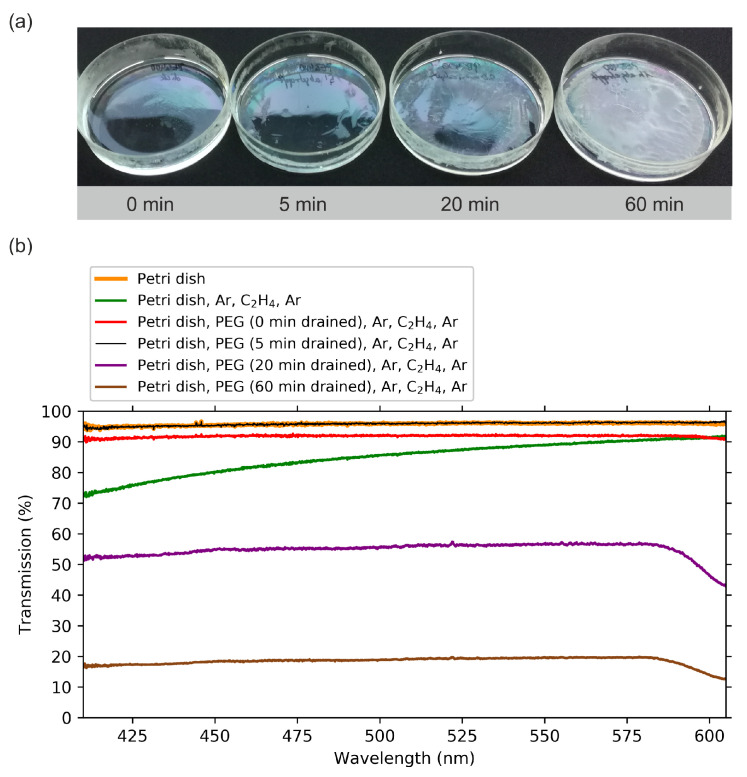
(**a**) Photographs and (**b**) optical transmission spectra of plasma-coated PEG held in Petri dishes for different draining durations. There was no PDMS backing added and the samples had not yet been lifted off the Petri dishes.

In Figure 4 the effect of the draining duration on the optical transmission of PEG treated with the Ar-C${}_{2}$H${}_{4}$-Ar process is shown. In this case, the samples (Figure 4a) were still located in the Petri dishes when photographed and analyzed, and were not yet covered and lifted off with the PDMS backing. It is clearly visible that the matte effect of the plasma-treated surface was enhanced for a longer draining duration, which corresponds to a lower filling level of liquid PEG. This observation was confirmed by the optical transmission spectra displayed in Figure 4b. For comparison, two additional spectra are displayed: one of an empty Petri dish, another one of a dish coated with the same PPF as used for the deposition on liquid PEG. The drop in transmission towards lower wavelengths for the coated dish is in accordance with the observed brownish appearance of such coatings with the applied thickness of 200 nm.

The spectra of the samples for which the PEG had been drained for only 0 and 5 min show a slight reduction in transmission caused by the PEG layer. This corresponds with the photographs of the samples where we observe an inhomogeneously distributed cloudiness on the surface. Again, when inspecting the spectra in Figure 4b, one has to remember the circumstance that the resulting intensity of the transmitted light depends on the region where it was measured. That can explain why the signal of the sample drained for 5 min overlaps with the spectrum of the empty Petri dish and is thus slightly higher than the one of the sample which had not been drained at all. A significant effect, however, can be seen for the 20 and 60 min drained samples, which reduced the transmission to 57% and 19%, respectively. The photographs show the surface closing more and more with increasing draining duration, and the optical spectra confirm the enhanced matte effect. These results suggest that the process of successful film formation critically depends on the thickness of the treated PEG film and that a PEG film not thicker than 20 to 30 μm is necessary to obtain a closed surface.

To identify possible reasons for this effect, the influence of the distance between the substrate surface and electrode of the plasma reactor during film formation was investigated. Higher filling levels of the PEG substrate increase this distance, leading to a slight reduction in the thickness of the PPF. However, deposition on solid substrates at varying distances from the electrode showed that for a distance corresponding to the highest PEG filling level in the range of ∼170 μm, the PPF thickness is reduced by only 1%. Since a successful covering of thin PEG films with a closed, matte surface could be achieved when reducing the thickness of the PPF to 100 nm and even to 50 nm, the effect of changing PPF thickness on the resulting sample surface can be neglected. The filling level may, however, affect the morphology of the PPF and could thus have an influence on film formation, as will be discussed in the following section.

### 2.2. Surface Morphology

Measurements via optical, confocal laser scanning (CLSM) and scanning electron microscopy (SEM) were conducted to get insights into the surface morphology of the samples. During the plasma process, more or less closed films formed on the liquid PEG substrates. Those films were studied using optical microscopy and CLSM. Afterwards, they were covered and stabilized with PDMS. The actual sample surface became accessible by lifting off the samples. Those surfaces were studied via CLSM and SEM imaging.

**Figure 5:**
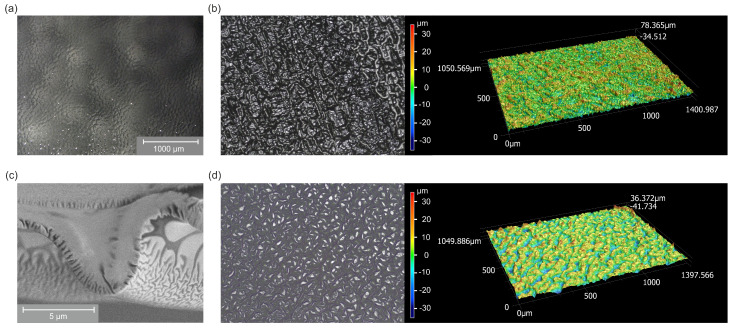
(**a**) Optical and (**b**) CLS microscopy images of a PEG substrate coated with a C${}_{2}$H${}_{4}$ plasma and drained for 60 min (both without PDMS backing, not yet lifted off the Petri dish). (**c**) Scanning electron and (**d**) CLS microscopy images of an Ar-C${}_{2}$H${}_{4}$-Ar sample (both with PDMS backing, lifted off the Petri dish).

The inhomogeneity of the optical appearance of the Ar-O${}_{2}$ treated PEG samples, shown in Figure 2a, could be confirmed by optical microscopy and CLSM. For different spots on the samples, very smooth and wrinkled areas with strong variations in surface roughness could be found. For the Ar-C${}_{2}$H${}_{4}$-Ar samples a more reliable determination of surface roughness was possible due to more homogeneous surfaces. Figure 5a displays an optical microscopy image of PEG treated with Ar-C${}_{2}$H${}_{4}$-Ar plasma. The picture was taken from the PPF deposited onto the liquid PEG substrate drained for 60 min before covering this surface with PDMS and before lifting the sample off the Petri dish. Therefore, the structure becoming visible represents the original state of the PPF’s morphology as it forms when being deposited onto the liquid. A bumpy surface structure appears homogeneously distributed over the areas where film uniformity could be expected according to optical observations. The lateral size of those structures is in the range of 100 μm. CLSM imaging revealed the extension of these features in the vertical direction and permitted us to quantify the surface roughness of the PPF. A roughness value $R_{a}$ = 4.8 μm (arithmetical mean deviation) and a maximum height between the lowest and highest value of the profile of $R_{z}$ = 78 μm (Figure 5b) were measured. When lifting off the samples and looking at the surface covered with PEG, again, uniform buckling could be observed (Figure 5d). The roughness values were in a similar range as before lifting off the samples ($R_{a}$ = 5.7 μm, $R_{z}$ = 69 μm).

Those measurements were repeated for samples with different PEG draining durations. The PPFs coated onto liquid substrates with different filling levels showed roughness *R*${}_{a}$ between 3.4 and 8.8 μm and maximum profile heights *R*${}_{z}$ in a range between 40 and 100 μm. These values did not show a clear correlation with the draining duration, but rather, depended on the surface area where the values were measured. Especially for shorter draining durations, no or only small spots could be found which featured buckling due to successful film formation. Those areas showed similar structural properties, as did the closed films for long draining periods.

In addition to the surface morphology of the PPF just described, SEM recordings of samples which were lifted off reveal that these features are overlaid by even smaller ones on the PEG side. The image in Figure 5c was taken by looking at the cut surfaces. The large-scale curling of the sample is caused by the preparation process. The upper part of the image shows the homogeneous area of the PDMS backing and the plasma coating. Underneath, pointing towards the lower edge of the image, smaller, wrinkled features distributed across the whole surface are visible. Their extension is in the order of 100 nm; their depth ranges from roughly 500 nm to 1 μm.

### 2.3. FTIR Spectroscopy

**Figure 6:**
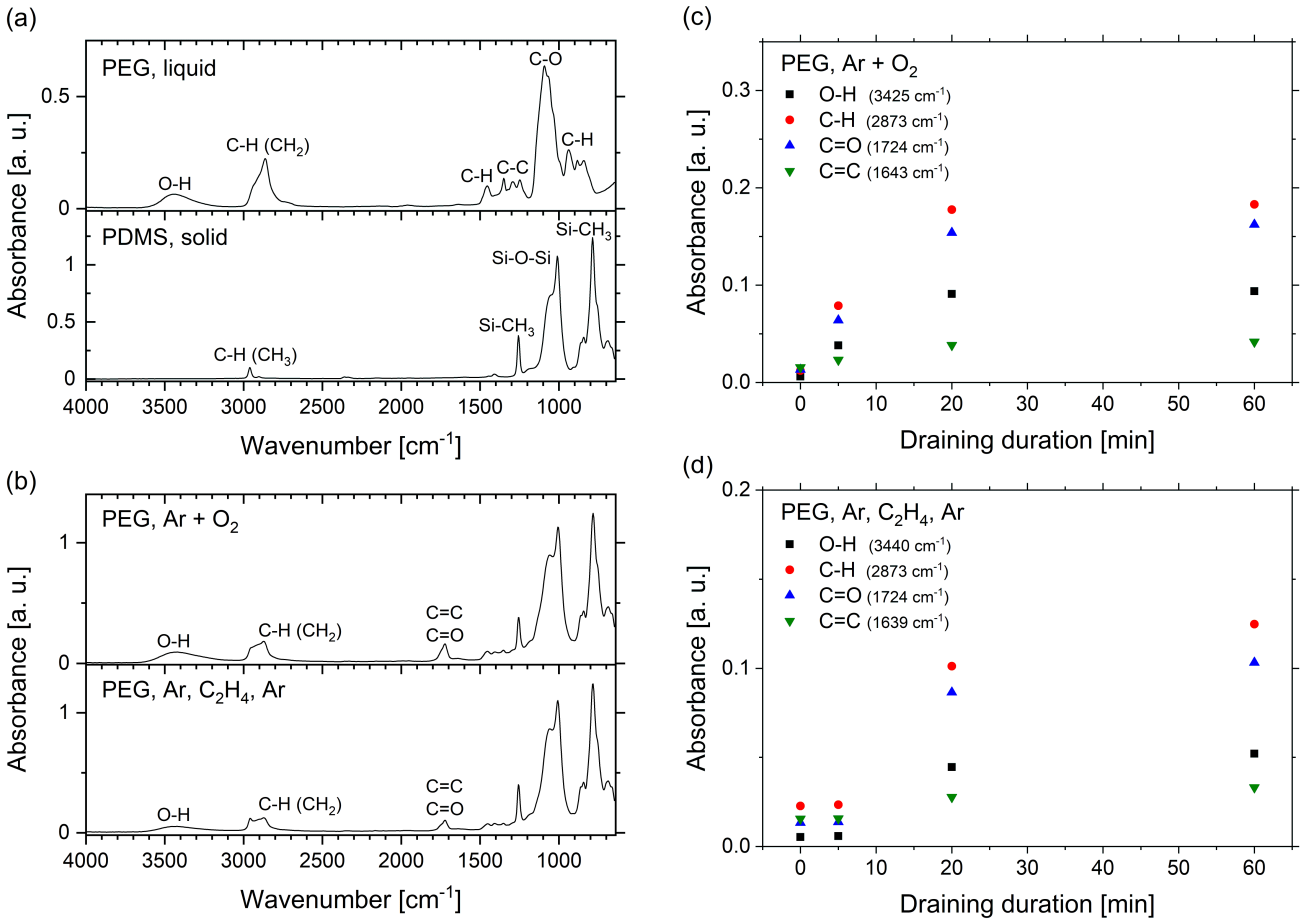
(**a**) FTIR spectra of liquid PEG and cured PDMS. (**b**) FTIR spectra of plasma-treated PEG samples. (**c**) Peak intensity of the Ar-O${}_{2}$ samples depending on the draining duration. (**d**) Peak intensity of the Ar-C${}_{2}$H${}_{4}$-Ar samples depending on the draining duration.

To get an insight into the composition of the surfaces created by plasma processing of liquid PEG substrates, FTIR spectra were recorded. Figure 6a shows the spectra of liquid PEG and the solid PDMS backing as references. The PPF can be neglected, since it contributes only weakly to the signals of C–C and C–H bonds, which overlap with corresponding strong bands of PDMS and PEG. PEG shows a typical absorption band between 3100 and 3650 cm${}^{-1}$, which corresponds to the O–H stretching vibration. This is one of the main features which can be used to distinguish PEG potentially attached to the sample’s surface from the PDMS backing. Another pronounced peak at 2862 cm${}^{-1}$ belongs to the asymmetric C-H stretching vibration of CH${}_{2}$ which differs from the C–H stretching of PDMS at the slightly higher wavenumber of 2962 cm${}^{-1}$ originating from CH${}_{3}$. Between 1420 and 1190 cm${}^{-1}$ the PEG spectrum features characteristic C–C stretching vibrations; and at 1454 cm${}^{-1}$ and between 968 and 755 cm${}^{-1}$, C–H bending vibrations can be found. The double band of the C–O stretching vibration appears at 1060 cm${}^{-1}$ and 1092 cm${}^{-1}$, close to the Si–O–Si stretching signals of PDMS at 1060 cm${}^{-1}$ and 1010 cm${}^{-1}$. The peaks at 1257 cm${}^{-1}$ (symmetric stretching vibration) and 787 cm${}^{-1}$ (rocking vibration) in the PDMS spectrum indicate the Si-CH${}_{3}$ bond.

These reference spectra can be compared with the spectra taken of the plasma-treated and coated PEG samples in Figure 6b in order to verify the presence of PEG on the examined surfaces. Both the Ar-O${}_{2}$ and the Ar-C${}_{2}$H${}_{4}$-Ar samples show the characteristic PEG signals of the O–H bond (3650–3100 cm${}^{-1}$) and the C–H bond (2862 cm${}^{-1}$). In comparison to the spectrum of pure PEG, the ratio between the C–H and O–H vibrations is reduced for the plasma-treated/coated PEG samples. Instead, two additional bands appear at 1724 cm${}^{-1}$ and 1639 cm${}^{-1}$ which can be assigned to the C=O and the C=C stretching vibrations. The presence of these double bonds can be associated with a partial cross-linking of the original PEG molecules during the plasma process, but may partially also be caused by oxidative degradation when exposed to oxygen and light after the process [68,72,73,74,75].

The characteristic PEG signals in the spectra of the samples confirm that PEG had successfully been attached to the surface by both the Ar-O${}_{2}$ and Ar-C${}_{2}$H${}_{4}$-Ar plasma processes. The spectra for the samples treated with argon and oxygen seem to feature more pronounced PEG signals than the ones of the Ar-C${}_{2}$H${}_{4}$-Ar samples. This can be explained by the fact that the spectra were normalized to the Si–O–Si peak of PDMS at 1010 cm${}^{-1}$. The PPF in the Ar-C${}_{2}$H${}_{4}$-Ar samples increases the distance between silicone backing and substrate surface, thereby leading to a weakening of this signal. Therefore, the comparison of the intensities of the spectra does not provide quantitative information about the presence of PEG on the two different types of samples.

To see if different filling levels of liquid PEG do not just affect the optical properties of the samples but also change the attachment of PEG to the surface, FTIR spectra were recorded for samples prepared by applying different draining durations, resulting in different heights of the original PEG substrate. Instead of displaying the whole spectra, we focus on the significant peaks only, which correspond to the O–H, C–H, C=O and C=C bonds. Figure 6c,d shows the maxima of these peaks for the Ar-O${}_{2}$ and the Ar-C${}_{2}$H${}_{4}$-Ar samples. For draining durations of 20 and 60 min the PEG signals increase significantly compared to the shorter time periods. This correlates with the decreasing filling level of the PEG substrates (Figure 3) and leads to the conclusion that a PEG layer with a maximum height between 20 and 30 μm is a critical requirement for a successful covering and attachment of PEG molecules to a plasma-polymerized film by the presented top-down approach. Furthermore, these results show that the optical properties of the samples, which were explained above, in fact correlate with the presence of PEG on the samples’ surfaces.

### 2.4. XPS Measurements

**Figure 7:**
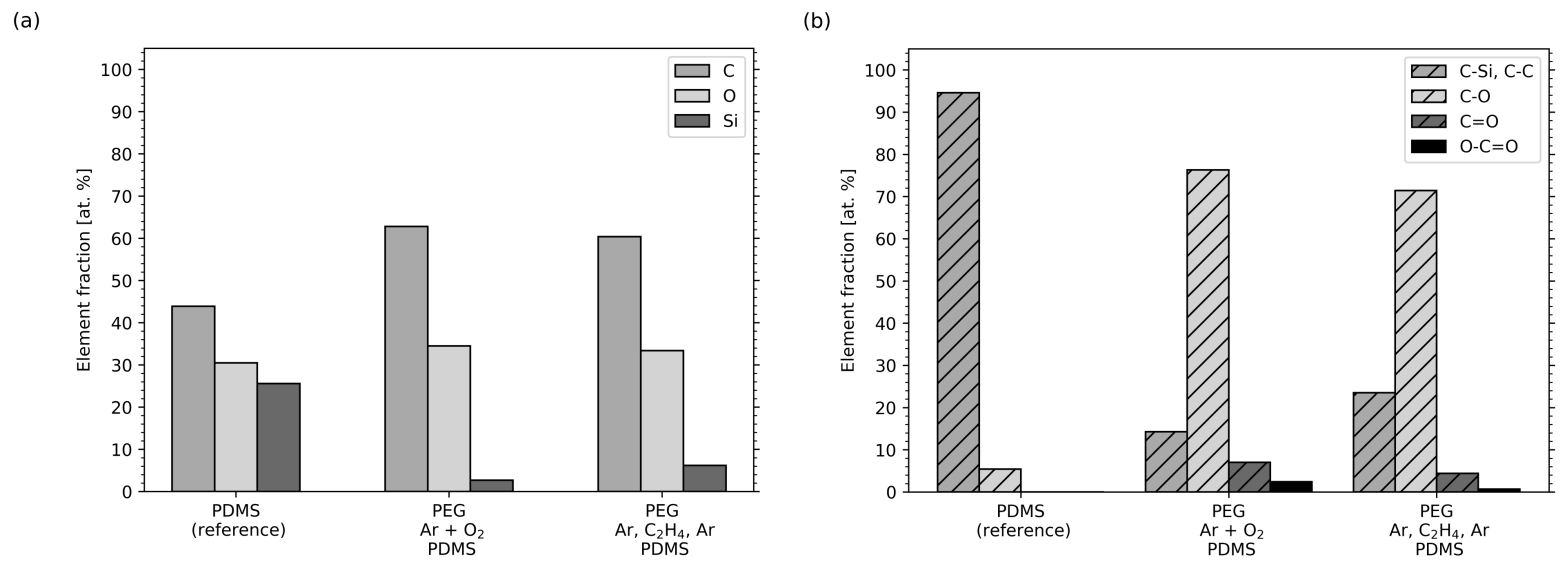
Elemental composition of PDMS and plasma-processed PEG obtained from XPS region scans. The values have been normalized to 100%. (**a**) Single elements and (**b**) chemical compounds.

**Figure 8:**
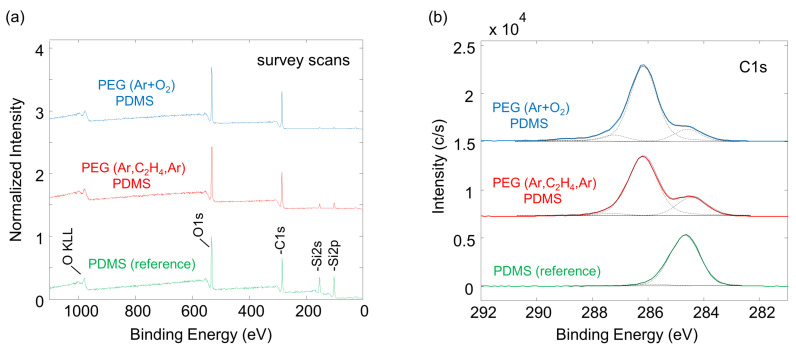
Comparison of XPS spectra of PDMS and plasma-processed PEG. The spectra are offset in the *y*-direction for better visualization. (**a**) Survey scans with a normalized signal intensity of one for the strongest peak. (**b**) Carbon C1s high-resolution scans (full lines) and composite peaks.

To obtain additional information about the composition of the samples, XPS measurements were performed at the surface of the PEG attachment. In Figure 7 the results for the Ar-O${}_{2}$ and Ar-C${}_{2}$H${}_{4}$-Ar samples are compared, and Figure 8 depicts the corresponding XPS survey and high-resolution spectra.

Figure 7a shows the content of the elements present in the samples. Only the elements carbon (C), oxygen (O) and silicon (Si), as expected for PDMS and PEG, were clearly observed (see Figure 8a). No other elements were present on the surface. As a reference, pure, cured PDMS used as silicone backing for the samples was examined. It showed 43.9% carbon, 30.5% oxygen and 25.6% silicon. The surface chemistry for the two samples with a PEG functionalization significantly changed from the corresponding PDMS reference sample. In particular, a change in the C/O ratio and a significantly reduced silicon concentration was observed, pointing towards successful PEG attachment to the surface in both cases.

In Figure 7b the different carbon chemical compounds are displayed, and Figure 8b shows the corresponding high-resolution elemental scans for carbon. Considering the molecular structure of PDMS, carbon is mostly present in the form of C–Si bonds (reference binding energy at 284.6 eV) plus some minor part of C–C/C–H and C–O adventitious carbon contamination. C–Si and C–C/C–H bonds were taken together as one component in the fitting because there is no significant binding energy shift between these two bands [76]. The C–O bond is weak in the PDMS reference sample, ruling out significant adventitious carbon contamination. For the Ar-O${}_{2}$ and the Ar-C${}_{2}$H${}_{4}$-Ar samples, significantly increased intensities for the single oxidized carbon C–O (285.8–286.6 eV) and additional bonds corresponding to multiply oxidized carbon, i.e., C=O (287.2–288.2 eV) and O-C=O (288.8–290.0 eV), have been assigned (see Figure 8b). Thereby, the component peak energy was restrained to those binding energy ranges, which are taken from literature values [76]. The full width at half-maximum (FWHM) was fixed, as is customary for the analysis of plasma polymer films [77].

For the Ar-O${}_{2}$ sample the measured, silicon content was reduced to 2.7%, whereas the fractions of carbon and oxygen increased to 62.8% and 34.5%, corresponding to an increase in the C–O signal (C-O is 76.3% of the total carbon signal; see Figure 7b). Small fractions of C=O and O-C=O appear as well, which confirms the partial cross-linking of PEG and post-process oxidation, as was already discussed when evaluating the FTIR spectra. When looking at the results of the Ar-C${}_{2}$H${}_{4}$-Ar sample, the findings are similar. The carbon content increases to 60.4% and the amount of oxygen to 33.4% compared to the reference sample. Again, this result is accompanied by an increasing number of C–O bonds (71.4% of the total carbon signal). The silicon content was reduced to 6.2%. The C–O bond is typical for PEG [76] and further proves the PEG layer covering the PDMS for both types of samples. As mentioned, small amounts of silicon were present on the surfaces of the Ar-O${}_{2}$ and Ar-C${}_{2}$H${}_{4}$-Ar samples. Most likely, it originated from the PDMS backing. Since XPS is a surface sensitive method, the silicone must have reached to the surfaces of the samples in order to be measurable. It has been reported that PDMS contains low-molecular-weight molecules that can easily diffuse through a 50 to 70 nm thick carbon coating within hours and restore hydrophobicity of a former hydrophilic surface [78]. Thus, this diffusion can be assumed to have taken place in our samples, since it can be inhibited but not completely prevented by the PPF with a thickness of 200 nm, and the partially cross-linked PEG layer, which had a thickness in the same range, as we reported earlier [50].

### 2.5. WCA Measurements

The previous results clearly showed that there was PEG attached to the surfaces of the samples. To investigate how this affects surface properties, water contact angle measurements were conducted.

**Figure 9:**
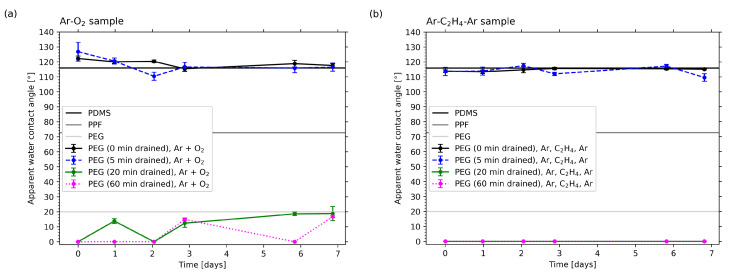
Temporal evolution of the apparent water contact angle on the (**a**) Ar-O${}_{2}$ and the (**b**) Ar-C${}_{2}$H${}_{4}$-Ar samples. For comparison the WCA values for pure PDMS, the plasma-polymerized film and PEG are drawn as straight lines.

In Figure 9a,b the apparent water contact angle values for the Ar-O${}_{2}$ and Ar-C${}_{2}$H${}_{4}$-Ar samples are plotted for different draining durations of the liquid PEG substrates, which were stored and measured over the period of one week. For comparison, the contact angles for pure PDMS (115.9°± 1.2°), the PPF (72.7°± 1.6°) and liquid PEG (20.0° ± 1.2°) were measured and are drawn as straight lines. For measuring the WCA on PEG, a thin layer of the liquid was applied to a glass slide. The values for PDMS and PEG correspond well with values known from literature [59,61,79,80]

Even if the applied plasma processes differ for the two types of samples, they are quite similar in the resulting surface properties concerning hydrophilicity. Again, the effect of draining duration on the successful attachment of PEG, as discussed earlier, becomes clearly apparent. While no draining at all and a short draining of 5 min do not show any effect, meaning that the contact angles for these samples are still in the range of the ones of pure silicone, the samples for which the PEG substrates had been drained for at least 20 min were highly hydrophilic, featuring contact angles even lower than those of PEG. For the Ar-O${}_{2}$ samples, values between 0° and 20° were measured. The variation of values during the measuring period of one week originated from the non-uniform surfaces of those samples. For each time step, five measurements on different spots of each sample’s surface were conducted. However, those spots were not necessarily the same each time, leading to a slight variance. For the Ar-C${}_{2}$H${}_{4}$-Ar samples, contact angles were not even measurable, since the droplets did not only spread completely but were even absorbed by the PEG layer. These excellent hydrophilic properties did not change significantly during one week of storage for any of the samples. For longer storage times, the contact angles gradually increased, but even after five months, values between 50° and 70° were measured, indicating that the surfaces were still hydrophilic.

The finding that the contact angles slightly increase over time cannot be explained by hydrophobic recovery, as is described for common plasma-polymerized films [81,82]. That effect originates from the fact that the surface is brought to a non-equilibrium state by plasma treatment and later returns to equilibrium by migration and reorientation of polar groups. For the presented top-down approach, however, it can be assumed that the PEG itself forms a quite thick layer, and that therefore only its backside is in direct contact with the plasma for a short period of time [50]. Due to this shielding effect, no additional carbon atoms are incorporated into the PEG layer near its surface (which is pointing downwards during the preparation process). Thus, the C/O ratio and with it the polarity and degree of hydrophilicity can be assumed to stay close to the one of the starting material PEG. A more plausible explanation for the gradual loss of functionality is a combination of oxidative degradation, which is known to take place in grafted PEG surfaces and the aforementioned diffusion of siloxane molecules from the PDMS backing to the sample’s surface, which alters the chemical composition and thus surface properties over time [72,75,78]. As the XPS results revealed the presence of silicon on the samples’ surfaces, it can be assumed that siloxane diffusion plays a mayor role in the loss of hydrophilicity.

**Figure 10:**
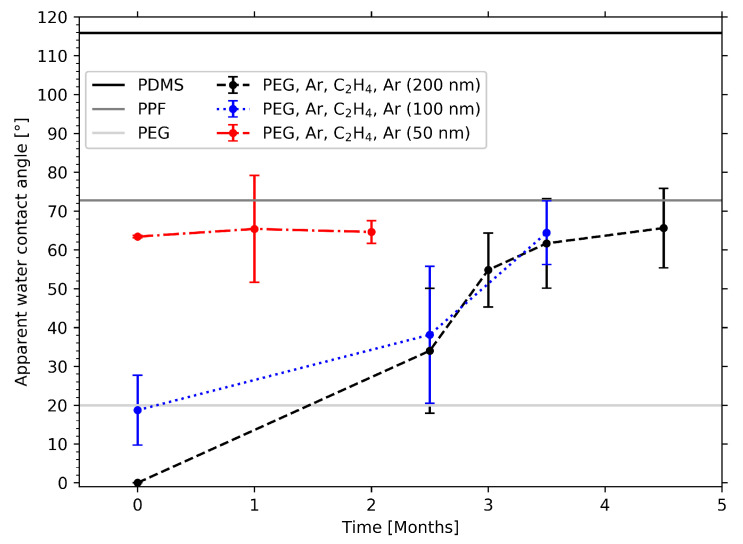
Temporal evolution of the apparent water contact angles on the Ar-O${}_{2}$ samples and the Ar-C${}_{2}$H${}_{4}$-Ar samples with plasma-polymerized film (PPF) thicknesses of 50, 100 and 200 nm. For comparison, the WCA values for pure PDMS, the plasma-polymerized film and PEG are drawn as straight lines.

To provide further proof for this claim, the thickness of the PPF was varied. Films with thicknesses of 50, 100 and 200 nm were deposited on liquid PEG as before, and contact angles were measured on the resulting PEG surfaces: once, after the samples had been lifted off, and again, multiple times over a period of several months. The resulting values in Figure 10 demonstrate that all three samples were hydrophilic with contact angles < 70°. If large amounts of silicone were present on the samples’ surfaces, much higher contact angles would be expected. However, an increase in WCA values can indeed be observed with decreasing film thickness for the freshly prepared samples. As shown before, the water droplet got completely absorbed by the PEG layer of the Ar-C${}_{2}$H${}_{4}$-Ar sample with a 200 nm thick PPF. In the graph this is indicated by a WCA of 0°. The contact angle of the sample with a PFF of 100 nm thickness already features a WCA of about 20°. The value further increases to 63° for the 50 nm sample.

This dependence of the WCA on film thickness can be explained by the fact that the length of the diffusion path increases for thicker PPFs. Measuring the WCA directly after preparing the samples does not yet allow diffusion of silicone through the thickest PPF leading to a highly hydrophilic surface. The surface wettability of the samples with the thinner PPFs, however, is already affected. With increasing diffusion time the longer distance through the thicker coatings can also be overcome. This is illustrated by the WCA values measured after several months. The values for the samples with the 100, and 200 nm PPFs increase and after 3.5 months their wettability reaches values between 60° and 70°, which is comparable with the WCA that the 50 nm sample showed from the beginning. This suggests that these values represent a stationary state and that a saturation of siloxane molecules on the samples’ surfaces is reached.

## 3. Discussion

With the results presented above we confirmed that it is possible to form solid films on the surface of liquid PEG by either cross-linking the liquid’s molecules via an Ar-O${}_{2}$ plasma or by depositing a PPF using an ethylene plasma. Furthermore, it could be shown that those low-pressure processes enable covalent bonding of PEG molecules to those films.

Optical inspection and results of OTS measurements of the Ar-O${}_{2}$ samples show a quite strong non-uniformity which can be explained by the stabilization process. The PEG film may not be stable enough along the whole surface area to withstand the coverage with liquid silicone. As a result, silicone from the backing might penetrate through the weaker cross-linked regions and displace the PEG. Hence, the cross-linked, matte PEG surface is not homogeneously distributed on the resulting sample. In contrast, the a-C:H film deposited on the liquid PEG results to form a stable barrier for the silicone backing leading to more uniform sample surfaces.

The investigation of the PPF deposited onto the liquid revealed that a successful formation of stable films and an attachment of PEG critically depends on the thickness of the liquid substrate material. OTS of the liquid PEG substrate only covered with the PPF showed that short draining durations corresponding to PEG filling levels > 50 μm lead to mostly transparent or only partly and non-uniformly covered surfaces whereas longer draining causes formation of a closed matte film. It can be assumed that the mobility of solid clusters forming on the liquid’s surface and their potential to submerge in the liquid are enhanced when depositing on thicker PEG layers. This may inhibit the formation of closed films. When deposited on a solid substrate the PPF is known to be transparent. The observed inhibition of light transmission for the solid-on-liquid samples can therefore be attributed to the presence of surface morphology and an additional PEG layer attached to the samples lifted off the Petri dish. FTIR results of those sample surfaces clearly indicate the presence of PEG for longer draining durations and reveal that the height of the PEG substrate does not only affect film formation itself but at the same time the successful attachment of PEG to the film. The change in optical appearance of the samples with draining duration can thus directly be correlated with the degree of PEG attachment. This becomes manifest in the surface wettability determined by WCA measurements. The results show that longer draining and with it a thinner PEG film leads to highly hydrophilic surfaces due to the presences of PEG, whereas thicker PEG substrates inhibit a surface functionalization. Contact angle measurements also indicate that for short draining durations there is not only no PEG attached to the sample’s surface but that not even WCA values of around 73° are reached which would point to the presence of the PPF. It can be assumed that, similar to the case of the Ar-O${}_{2}$ samples, the film formed on those relatively thick PEG substrates was not able to sufficiently support the PDMS backing. With the a-C:H film breaking and submerging in the liquid substrate the silicone was the only material left at the sample’s surface after the curing and rinsing procedure resulting in corresponding WCAs around 115°.

To understand why the process of film growth and attachment of PEG critically depends on the thickness of the treated PEG substrate it’s necessary to take a look at the role of film morphology. We could show that the PPFs deposited onto liquid PEG substrates and the resulting PEG surfaces of the samples lifted off the Petri dishes feature a pronounced surface topography. The observed deformation of the otherwise flat film occurs during film formation due to inner film stress. On a solid substrate such stress leads to crack formation or buckling of the film depending if the internal stress first exceeds the cohesive forces inside the PPF or the adhesive forces between PPF and substrate [83,84,85,86]. For liquid substrates coated with solid films the appearance of uniformly distributed structures has been reported and linked to the internal film stress which is released via deformation since the liquid substrate can follow the wrinkling film [1,6,10,15,46,87]. Appearance and dimensions of the surface features appearing on our samples are very similar to what was observed by those authors for different kinds of substrates and film-forming materials.

The measured surface roughness values help to understand the mechanism of film formation on the liquid and successful attachment of a PEG layer to the samples. The vertical extension of the wrinkling PPFs is in the range between 40 and 100 μm. Assuming that the buckling film moves the same distance downwards as it does upwards means that the wrinkles can reach between 20 and 50 μm towards the bottom of the Petri dish. On the other hand, it could be shown that a PEG film no thicker than 20– 30 μm corresponding to a draining duration of 20–60 min is necessary to obtain a closed surface equipped with covalently bond PEG. This leads to the conclusion that for thinner PEG substrates the PPF touches the bottom of the Petri dish in certain spots which correspond to the minima of the surface profile. This circumstance in turn seems to be crucial to stabilize and support the PPF keeping it from sinking in the PEG substrate and making it resistant to the compression resulting from the silicone backing. As the lateral distance between those buckles is in the range of 100 μm there is enough space in between to be filled with PEG which is bond to the PPF’s surface providing the observed surface properties.

The long-term stability of the functionalized surfaces could be proven by WCA measurements showing that there was no significant change in hydrophilicity during the first week of sample preparation. For longer storage times WCA values increased gradually up to 60°–70°, most probably due to the diffusion of siloxanes from the silicone backing. But the samples did not loose their hydrophilicity even after several months which makes them superior to optimized functional plasma polymer films [88]. Other groups using different methods to prepare samples equipped with PEG also reported contact angles staying below 50°–70° after storage periods of several weeks [89,90]. However, WCAs already started to increase towards those values after several hours or days. Compared to that, our surfaces exhibit the ability to ensure complete spreading of water droplets and even water absorption for at least one week. The durability may even be further enhanced by optimizing the PPF thickness and the method for stabilizing and taking off the sample in order to cause fewer cracks and thus a reduced siloxane diffusion over time. In addition, our approach has the huge advantage of being sustainable and less complex compared to other methods, such as the grafting of PEG brushes which are time consuming and use complex wet chemistry techniques requiring multiple process steps [54,55,59].

The highly hydrophilic and even water absorbing surface properties of the presented samples are of special interest for biological and biomedical applications for which hydrophilicity and the formation of a hydration layer is a crucial requirement leading to pronounced protein resistance and anti-fouling properties [52,53,54,55,56,57,58,59,60,61,62]. Furthermore, surface topography has been found to be another crucial factor for inhibiting biofouling [91]. This makes the presented approach even more interesting with its potential to simultaneously control surface functionality and morphology leading to specifically designed surfaces. Being able to adjust surface morphology may also be advantageous for the production of flexible electronics and sensors [92,93,94,95,96]. In addition, PEG surfaces are known to be lubricious [75,97,98,99] which is why further development of our functional surfaces towards low-friction applications is desirable. Furthermore, the presented top-down approach provides the possibility to produce functional surfaces on various kinds of geometric structures by simply using an appropriate mold for holding the liquid PEG substrate. In this way channels and other microfluidic structures can easily be equipped with a hydrophilic surface preventing biofouling which is a serious problem in the biomedical field [61,100,101].

Such applications can be successfully implemented in the future by further optimization of the film forming and cross-linking process. To this end it will be necessary to study the influence of PPF thickness on the resulting surface structure in more detail. Another challenge will be the understanding of factors influencing surface homogeneity. Since the Petri dishes holding the PEG have a 3D geometry, the transport of film-forming plasma species to the substrate surface is even more complex than for simple flat substrates. Surface uniformity can be assumed to be affected by the influence of the rim of the Petri dish, since it causes a shadowing effect posing a barrier for the flow of monomer gas and film-forming radicals. A suitable tool to analyze this effect and to improve surface homogeneity is a numerical simulation model of the transport of film-forming material as we presented it earlier [102].

## 4. Materials and Methods

### 4.1. Materials and Sample Preparation

The liquid used as substrate material for the experiments is PEG-400 (Sigma-Aldrich). It is a transparent fluid with a viscosity of 92.8 mPa·s [71] and a low vapor pressure (< 0.1 hPa [103]). For the sample preparation empty Petri dishes (diameter: 54 mm, height: 12 mm) were treated with an Ar-O${}_{2}$ plasma (2 min, 20 sccm Ar, 5 sccm O${}_{2}$, 50 W, 0.1 mbar) to improve their wettability. This facilitated the spreading of liquid PEG of which 2.5 g were filled into each dish. Those substrates were turned until the opening of the Petri dish pointed downwards at an angle of about 35° and left for specific periods of time (5, 10, 20 and 60 min) which led to a partly draining of liquid. The draining procedure was conducted under vacuum conditions in order to remove any residual gas which may had been entrapped in the liquid when pouring the PEG and to avoid the formation of bubbles during the subsequent plasma process. For the plasma treatment and coating, respectively, a symmetric, capacitively coupled reactor was used which is described in more detail elsewhere [104,105]. For sample preparation two different parameter sets were applied which can be found in Table 1. Before depositing the PPF from an ethylene plasma an argon plasma pre-treatment step caused a slight cross-linking and improved attachment of PEG molecules to the film. The second process step was started at a pressure of 0.2 mbar to provide mild deposition conditions comprising low ion energies at the liquid-solid interface, followed by a reduction in pressure to 0.1 mbar to enhance the cross-linking during film growth [106]. The depositing step was followed by another argon plasma treatment to improve the adhesion between PPF and silicone backing.

Plasma processing caused the formation of a film on the PEG’s surface already visible with the naked eye. However, those films were too thin and fragile in order to being lifted off the Petri dish allowing to access the lower side of the film where PEG was supposed to be attached. As a solution to this problem the backside of the film was stabilized with a silicone layer. For this backing 3.3 mL PDMS (Dowsil 184, Silicone Elastomer Kit, Dow Europe GmbH) was chosen which allowed to distinguish between functionalized surface and backing when analyzing the samples. The mixing ratio of PDMS and curing agent was 10/1. Here, the solid film formed on the liquid substrate did not only cause a cross-linking and attachment of PEG molecules but also served as a diffusion barrier between silicone and PEG. For curing the PDMS the samples were placed in an oven with a constant temperature of 60 °C for three hours. After that the samples could be lifted off the Petri dish revealing the PEG side. Remaining PEG which was not bound to the surface was removed by rinsing the samples with deionized water. The step-by-step process of sample preparation is displayed in Figure 1.

### 4.2. Analytical Methods

To measure the optical transmission of the samples we used a fiber-optically coupled spectrometer (USB2000, Ocean Optics). A 200 μm multi-mode fiber (NA 0.22, Thorlabs M92L) was used for the coupling of the light. As a light source a halogen cold light (FOT 150, FiberOptic P. + P. AG) was used. The samples were placed in a distance of 4 mm from the optical fiber. The sample’s surface morphology had been analyzed by using an optical microscope (VHX-1000D, Keyence), a confocal 3D laser scanning microscope (CLSM) (VK-X1000, Keyence) and a scanning electron microscope (SEM) (S-4800, Hitachi). For optical and CLS microscopy the sample surface could directly be analyzed. For SEM imaging the plasma-coated PEG samples were additionally sputter-coated with a thin gold-palladium film (40 nm) before covering them with liquid PDMS in order to provide sufficient contrast and being able to distinguish the silicone from the a-C:H film during the measurements. Afterwards the samples were cured and rinsed, as described before and subsequently cut to obtain a cross-section. The wrinkling of the PPF is represented by the roughness parameters S${}_{a}$ (arithmetical mean deviation) and S${}_{z}$ (maximum height between lowest and highest value) determined via CLSM using the software MultiFileAnalyzer version 2.2.0.93 (Keyence). To measure surface roughness also on less uniform samples areas with a surface structure representative for the largest portion on the sample’s surface was chosen. FTIR spectra of the samples were recorded with an FTIR spectrometer (640-IR, Varian) in attenuated total reflectance (ATR) mode in a wavenumber range between 4000 and 400 cm${}^{-1}$.

To determine the surface chemical composition of the samples, XPS measurements were performed with a VersaProbe II spectrometer (Physical Electronics, Chanhassen, MN, USA) using monochromatic Al Kα radiation (1486.6 eV) and a photoemission take-off angle of 45° with respect to the surface plane. The operating pressure of the XPS analysis chamber was below $1\times 10^{-6}$ Pa for all data presented here. Survey scan spectra (0–1100 eV) were acquired with an energy step of 0.8 eV, an acquisition time of 160 ms per data point and an analyzer pass energy of 187.85 eV. In addition, detail spectra of C 1s (278 eV to 298 eV), O 1s (523 eV to 543 eV) and Si 2p (94 eV to 114 eV) were recorded using an energy step of 0.125 eV, an acquisition time of 2.4 s per data point and an analyzer pass energy of 29.35 eV. The energy resolution (FWHM, full width at half-maximum height) measured on the silver Ag 3d${}_{5/2}$ photoemission line is 2.2 eV (for a pass energy of 187.85 eV) and 0.7 eV (for a pass energy of 29.35 eV). Total acquisition times were approximately 5 min for survey scans and 30 min per set of elemental scans. The samples adhered to a stainless steel holder via double-sided adhesive tape. Randomly chosen measurement surface positions were analyzed using a micro-focused X-ray beam of diameter 100 μm (operated at a power of 25 W at 15 kV). The 180° spherical capacitor energy analyzer was operated in the fixed analyzer transmission (FAT) mode. Sample charging was compensated using dual beam charge neutralization with a flux of low energy electrons (∼1 eV) combined with very low energy positive Ar ions (10 eV). The binding energy is referenced to the known binding energy of the C-Si signal (C 1s peak in polydimethylsiloxane at 284.6 eV [107,108,109]). Intensity determination and curve fitting was carried out with CasaXPS software version 2.3.16 (Casa Software Ltd, Teignmouth, UK) using a fixed 70% Gaussian, 30% Lorentzian product function to fit the XPS spectra. Atomic concentrations were calculated from XPS peak areas after subtracting a Shirley type background. Thereby, tabulated PHI sensitivity factors [110], corrected for our system’s transmission function and analyzer asymmetry parameter (correction due to a different angle between X-ray source and analyzer) have been used for quantification. Relative uncertainties in the measured concentration are estimated to be approximately ± 10%; the error estimate includes uncertainties in the background determination from the energy window setting and transmission function correction.

To study the wettability properties of the samples, the apparent water contact angle (WCA) was determined using a Drop Shape Analyzer (DSA25, Krüss GmbH). For each measurement a droplet of ultrapure water (270733-1L, Sigma Aldrich) with a volume of 2 μm was put on the surface. The spreading of the droplets was observed and the stationary contact angle value was measured after two minutes using the software Krüss Advance version 1.11.3.29401 in the Sessile Drop mode. For each sample five measurements were conducted on different spots of the surface and used to calculate a mean value and the standard deviation. Often, droplets spread completely and were even absorbed by the surface. Those measurements are indicated by a contact angle value of 0°. During and between the single WCA measurements the samples were kept under stable conditions of 60% relative humidity and 23 °C.

## 5. Conclusions

By using a low-pressure plasma process, we produced stable films on liquid polyethylene glycol (PEG). Depending on the process gas, either cross-linking of PEG or deposition of plasma-polymerized films (PPFs) on the liquid’s surface was obtained. In contrast to previous studies, the use of a plasma polymerization process enabled us to not only deposit thin films on liquid substrates but to attach them to the substrate material, resulting in a functionalized solid-liquid gradient. To stabilize those films, they were each covered with a silicone (PDMS) backing. The plasma cross-linked PEG films exhibited low mechanical stability hampering the stabilization process, resulting in non-uniform sample surfaces. The deposited PPFs, however, were sufficiently stable and featured good barrier properties to support the silicone and to prevent siloxane migration from the backing to the PEG substrate. After curing the silicone, the samples could be lifted off, and their PEG-functionalized side was analyzed.

Studying the chemical compositions of the samples’ surfaces via FTIR and XPS measurements verified the presence of covalently bonded PEG. WCA measurements showed strong and long-lasting hydrophilic and water absorbing surface properties which open up great potential for future non-fouling or low-friction applications. Optical inspection of the samples and optical transmission measurements revealed a correlation between successful PEG attachment and a reduction in optical transmission. Interestingly, a reduction in thickness of the initial liquid PEG substrate led to an improved surface functionalization. Analysis of the surface topography of the PPFs showed a characteristic wrinkling with a certain surface roughness caused by the plasma treatment. This turned out to be crucial for film stability when applying the PDMS backing. From the results, it can be concluded that the liquid PEG layer has to be thin enough to allow formation of closed PPFs geometrically supported through the bottom of the Petri dish which is holding the liquid. Only in this way can sufficient support be provided for the application of the PDMS backing. In addition, we noted the dependence of surface wettability on PPF thickness and time, which led to the conclusion that a gradual siloxane diffusion from the sample’s backing to its surface was taking place. This finding underlines the importance of the PPF as diffusion barrier.

The presented approach might as well be transferred to other systems. Applying different backing materials could help to prevent the diffusion of hydrophobic species to the functionalized surface. A variation of the liquid substrate material can enable the production of a broad variety of specifically tuned surface properties.

## Figures and Tables

**Table 1:** Process parameters applied in the two plasma processes used to treat/coat the PEG substrates.

	Step	Gas (Gas Flow Rate [sccm])	Pressure [mbar]	Power [W]	Duration [min]
**Process 1**	1	Ar (40) + O${}_{2}$ (10)	0.1	50	20
**Process 2**	1	Ar (40)	0.1	50	10
	2	C${}_{2}$H${}_{4}$ (16)	0.2 → 0.1	30	1 + 9
	3	Ar (40)	0.1	50	15

## Abbreviations

The following abbreviations are used in this manuscript:

a-C:H film	amorphous hydrocarbon film
CLSM	confocal laser scanning microscopy
FTIR	Fourier transform infrared
HMDSO	hexamethyldisiloxane
(i)CVD	(initiated) chemical vapor deposition
OTS	optical transmission spectroscopy
PDMS	polydimethylsiloxane
PECVD	plasma-enhanced chemical vapor deposition
PEG	polyethylene glycol
PHEMA	poly(2-hydroxyethyl methacrylate)
PPF	plasma-polymerized film
RF	radio frequency
SEM	scanning electron microscopy
WTA	water contact angle
XPS	X-ray photoelectron spectroscopy
